# *Legionella pneumophila*-induced cell death: Two hosts, two responses

**DOI:** 10.1080/21505594.2017.1384527

**Published:** 2017-11-10

**Authors:** Ascel Samba-Louaka

**Affiliations:** Laboratoire Ecologie et Biologie des Interactions, Microbiologie de l'Eau, Université de Poitiers, UMR CNRS 7267, Poitiers, France

**Keywords:** *Acanthamoeba castellanii*, apoptosis, *Legionellapneumophila*, macrophages, pyroptosis

*Legionella pneumophila* is a Gram-negative bacterium responsible of Legionnaire's disease, a severe form of pneumonia. *L. pneumophila* resides within natural and man-made aquatic systems. It shares these habitats with protozoa such as free-living amoebae that feed on bacteria. After uptake by amoeba, *L. pneumophila* is able to resist intracellular digestion and to multiply within this environmental host. Amoebae are considered as training ground for pathogenic bacteria such as *L. pneumophila*. Importantly, entry and intracellular replication of *L. pneumophila* within amoebae and mammalian macrophages display several similarities.^1^ Once engulfed, *L. pneumophila* avoids fusion of the phagosome with lysosomes and creates a favorable environment for replication, the replicative vacuole, which is surrounded by the endoplasmic reticulum and mitochondria.^2^ The ability of *L. pneumophila* to manipulate host cell functions is conferred by hundreds of effectors that are secreted or injected into the cytosol and the vacuole through type II (T2SS) and type IV (T4SS) secretion systems.^3,4^ Intracellular growth of *L. pneumophila* increases its resistance to antimicrobials facilitating dispersion of the bacterium.^5^

Although a number of mechanisms to infect amoebae and macrophages present common features and a shared evolutionary origin, some specificities were reported. For example, certain genetic loci allow *L. pneumophila* to infect macrophages, but not *Acanthamoeba*.^6^ In contrast, *L. pneumophila* mutants that are defective in inhibiting host translation have lowered growth in the amoeba *Dictyostelium* but show no replication defect in macrophages.^7^ Highlighting specific interactions between *L. pneumophila* and its different hosts is essential to understand the adaptation of *Legionella* to its multiple and evolutionarily distant hosts.

Another important issue is bacteria-induced cell death, which is relatively straightforward to observe, but difficult to characterize in detail. Difficulties come from the number of cell death pathways described to date. Bacteria could indeed induce cell death by activating apoptosis, pyroptosis, oncosis, necroptosis, NETosis, paraptosis or autophagic cell death.^8-10^ In amoebae, the absence of certain families of proteins such as caspases, renders the classification of the bacterial-induced host cell death arduous. In addition, bacteria can possess an arsenal of different effectors that either activate or repress the host cell death.

In this issue of Virulence, Mou and Leung address the expression of *L. pneumophila* genes that are involved in human monocyte and *Acanthamoeba* cell death.^11^ They also investigate the correlation of specific *L. pneumophila* gene expression patterns with the type of host cell death induced by the bacterium. The authors selected four set of genes, two which are involved in pyroptosis (*flaA* and *sdhA*) and two involved in apoptosis (*vipD* and *sidF*) of macrophages. The genes *flaA* and *vipD* encode respectively *L. pneumophila* flagellin and a phospholipase, and have been described to trigger host cell death.^12,13^ In contrast, *sdhA* and *sidF* are T4SS-translocated effectors that inhibit cell death.^14,15^

Mou and Leung point out differences in infection mechanisms depending on the host infected. Regarding genes related to pyroptosis, the authors observed a decrease of *flaA* expression and an increase of *sdhA* during infection of THP1 monocytes. Interestingly, the opposite result was obtained with the amoeba *Acanthamoeba castellanii*. Expression of *flaA* increased, while *sdhA* expression decreased. Expression of apoptosis-related genes *vipD* and *sidF* also differed during infections of both THP1 cells and *A. castellanii*. Infection of THP1 cells induced a decrease in mRNA levels of both *vipD* and *sidF*. In contrast, *A. castellanii* infection induced a very weak regulation of *sidF* and an earlier down-regulation of *vipD*, followed by a later up-regulation. Although the two genes involved in pyroptosis were differently expressed in *L. pneumophila* infecting THP1 and *A. castellanii* cells, they could promote a same phenomenon: repression of pyroptosis in monocytes or induction of cell death in amoebae. The biological relevance regarding the expression of apoptosis-related genes was less obvious, underlying the need to increase the panel of genes to study.

On the host side, the authors observed differences in uptake and replication of *L. pneumophila*. They found a higher replication of *L. pneumophila* in *A. castellanii* compared to THP1 cells. Moreover, the infection of monocytes with *L. pneumophila* was associated with a reduction of caspase-1 expression compared to uninfected cells. However, relationship between caspases expression and host cell death is not clear and need to be investigated. Overall, this study cumulated evidence suggesting that *L. pneumophila* could inhibit pyroptosis of THP1 cells.

The authors faced several limitations when they compared *L. pneumophila* infection of the two different hosts. For example, there is no evidence of the presence of caspases in amoebae, although caspase activities have been reported.^16^ Instead, caspase-like proteins (metacaspase or paracaspase) have been discovered.^17^ Despite similarities between caspases and caspase-like proteins, there are a few significant differences, such as the target cleavage site sequence.^17^ Another issue is that, in addition to the programmed cell death, metacaspases contribute to several cellular functions. Thus, the metacaspase-1 is involved in encystation of *A. castellanii*.^18^ Mou and Leung found that, over the incubation time, *L. pneumophila* induced an increasing metacaspase-1 expression, which was associated with the formation of cysts. This result has to be confronted with other studies showing that *A. castellanii* infected with *L. pneumophila* do not exhibit a cell wall containing cellulose as observed in mature cyst^19^ and present a low expression of metacaspase-1 at 48h post-infection.^20^

In summary, Mou and Leung demonstrated that expression of *L. pneumophila* genes involved in host cell death is diametrically opposite depending on the infected host. This study contributes to a better understanding of *L. pneumophila* adaptation to its very large spectrum of hosts. The expression pattern of pyroptosis-related genes in the environmental amoebal host could suggest the need of host cell death to ensure dissemination of *L. pneumophila*. In contrast, the control of monocyte cell death could be essential to maintain infection, as Humans are accidental dead-end hosts.
